# Bibliotherapy for stress management: a wellness intervention for first-year medical students

**DOI:** 10.5195/jmla.2025.1830

**Published:** 2025-04-18

**Authors:** Rebecca A. Morin, Amy E. LaVertu

**Affiliations:** 1 becky_morin@radcliffe.harvard.edu, Head of Research Services, Schlesinger Library, Harvard Radcliffe Institute, Cambridge, MA; 2 amy.lavertu@tufts.edu, Research and Instruction Librarian, Hirsh Health Sciences Library, Tufts University, Boston, MA

**Keywords:** Bibliotherapy, Wellness, medical education

## Abstract

**Objective:**

This proof-of-concept study aimed to evaluate if a library-initiated program of bibliotherapy could be effective in reducing overall levels of stress and anxiety in first-year medical students.

**Methods:**

This mixed-methods study consisted of an Interrupted Time Series (ITS) where participants established baseline levels of stress and anxiety by completing the 10-item Perceived Stress Scale (PSS-10) three times prior to intervention and three times following, with a bibliotherapy intervention delivered at the halfway point. Four focus groups were held following completion of the ITS with questions designed to solicit feedback related to how enjoyable and valuable participants found the study, as well as priorities for wellness.

**Results:**

An independent samples t-test was conducted to compare mean PSS-10 scores in the pre-intervention group to those in the post-intervention group. The results indicate no significant difference between scores pre-intervention (M= 17.85, SD=6.76) and post-intervention (M=17.21, SD=6.87, t(162)=.604, two-sided p=.547, 95% CI [−1.46, 2.75]). Focus group analysis revealed that participants found involvement in the study to be a useful component of a personal wellness or mental health maintenance program.

**Conclusions:**

Quantitative results did not achieve statistical significance, but analysis of focus groups indicates that participants derived benefit from involvement in the study, particularly related to the regular self-reflection required by completing the monthly PSS-10. The study is a successful proof-of-concept, indicating that medical students derive benefit from a librarian-led bibliotherapy program as part of student wellness.

## INTRODUCTION

In her memoir *White Coat: Becoming a Doctor at Harvard Medical School*, Ellen Lerner Rothman writes of a “collective doubt” that consumes her first-year classmates, arising from unspoken pressure to know everything, to ace every test, to heal every patient; yet in the midst of this shared experience, she notes, “we feared to acknowledge our private struggles, our perceived weaknesses” [1]. Over the last several decades, studies have shown that medical students struggle with “academic pressure, workload, financial concerns, sleep deprivation, [and] exposure to patients' suffering and deaths” [2]. As a result, the students exhibit “high emotional exhaustion, high depersonalization, and burnout” and a greater likelihood of depression and fatigue, when compared to other US college graduates aged 22–32 [3]. In response to these patterns, the Hirsh Health Sciences Library (HHSL) at the Tufts University School of Medicine (TUSM) launched a series of recreational programs called “Fun Labs,” offering students the opportunity to socialize and work on crafts. These activities were explicitly designed to be easy to stage and quick to complete. They required no preplanning and minimal time commitment, as lack of time and poor work-life balance are routinely reported stressors for medical students [4]. While popular and well-received, the ability to offer Fun Labs was halted by the COVID-19 pandemic in March 2020.

The pandemic and related mitigation measures are associated with increases in adverse mental health conditions and substance abuse in the general US population [5]. Health care workers endured additional stressors leading to high rates of burnout and mental health concerns including anxiety, depression, and insomnia [6]. Students in medical school in 2020 confronted additional challenges such as unexpected distance learning, social isolation, limited physical access to clinical settings and other students, as well as the fear of contracting COVID-19 and infecting others [7]. Recognizing that the abrupt changes and long-lived effects of the pandemic would increase stress and anxiety among students, we decided to explore the feasibility of starting a bibliotherapy program built on the principle that “information, guidance, and solace can be found through reading” [8].

Bibliotherapy, loosely defined as reading for therapeutic effect, has its modern roots in the Library War Service of the First World War. In collaboration with physicians, librarians “prescribed” books to soldiers recovering from illness or injury, to help boost the spirits and bolster the mental health of patients in military hospitals [9]. In the last 40 years, bibliotherapy has been successfully deployed in clinical settings, often utilizing a self-help text as a treatment or adjunct for conditions including depression, substance misuse, self-harm, panic disorder, and anxiety [10]. While there is awareness of bibliotherapy as a potentially useful tool in medical school settings, there is a lack of research focused on this population [11]. There is some evidence that students in health sciences disciplines respond positively to leisure reading or creative writing interventions which aimed to mitigate stress and anxiety, boost self-esteem, and improve overall psychological well-being [12], [13]. Research into the efficacy of bibliotherapy in the treatment of panic disorders has indicated that bibliotherapy combined with talk therapy is superior to bibliotherapy alone [14], while traditional therapy is vastly superior to any bibliotherapeutic intervention [15]. However, a 2010 trial postulated that in the absence of traditional therapy, having scheduled check-ins and limits may introduce a “deadline effect” that is motivational and could lead to improved patient outcomes [16]. In addition to panic disorder, bibliotherapy without regular therapist contact has shown improvement in cases of social anxiety disorder [17] and depression [18] as compared to no active intervention, although questions remain as to durability of treatment effects. Due to the intense demands on medical students' time and the physical distancing required due to COVID-19, a limited-contact model without additional meetings or talk therapy was adopted for this study. Crucially, as librarians we are neither able to diagnose or treat medical conditions, nor provide professional monitoring. We can, however, provide structure and deadlines.

We opted to replace the conventional self-help books used in many bibliotherapy studies with short readings, a mix of fiction and non-fiction selections from which participants could choose according to their interests. Use of non-fiction was supported by the traditional self-help texts used in bibliotherapy as well as research espousing use of memoir in the practice [11], [19]. Incorporation of fiction is supported by Dijikic et al's findings that reading fiction can lead subjects to develop “a decreased discomfort with ambiguity” [20]. This potential for increasing comfort with the unknown supported the selection of readings related to themes of plague and pandemic. Cognition researcher K.W.M. van Krieken theorizes that consumption of tragic/horror narratives enable readers to mentally prepare for challenging situations [21]. Anecdotally, this assertion appears to be supported by the 2020 reappearance of pandemic novels such as Stephen King's *The Stand* and Albert Camus' *La Peste* on best-seller lists, and the spring 2020 appearance of Stephen Soderbergh's 2011 film *Contagion* at the top of Netflix's most-streamed list [22]. Immersion in a horror narrative, whether via watching a film or television, listening, or reading, has been theorized to help the consumer build practical social skills and emotional resilience in the face of fear and chaos [23]. Building on this concept, this study aimed to determine if a self-paced, librarian-driven bibliotherapy model, in the form of excerpts of fiction and nonfiction related to plagues and pandemics, can serve as an effective intervention to improve perceptions of stress and anxiety among first year medical students.

## METHODS

### Study Conduct and Oversight

This mixed-methods study was conducted by two Research & Instruction Librarians (RM and ALV) at the Hirsh Health Sciences Library (HHSL) at the Tufts University School of Medicine (TUSM). The study protocol was approved by the Tufts University Social, Behavioral & Educational Institutional Review Board via Expedited means under 45 CFR 46.110 Categories 6 & 7 (IRB ID: STUDY00001125) in November 2020. Participants were recruited via the TUSM MD class of 2024 email listserv in December 2020. All aspects of the study were conducted electronically via Qualtrics and Zoom and administered by RM, and consent was obtained from all participants via a Qualtrics form. Quantitative analyses were performed by RM using IBM SPSS Statistics 28.0.1.0. Focus group recordings were saved to a University networked drive to safeguard data. Data were transcribed by RM using the MacOS Advanced Dictation tool. Zoom recordings were deleted from the local drive following review and transcription correction by both researchers. ALV completed initial hand-coding of focus group transcripts, followed by continuous comparison analysis conducted by RM using NVivo 1.6.2.

### Study Design

This study consisted of two parts: a quantitative data component structured as an Interrupted Time Series (ITS) and a qualitative data component composed of a series of small focus groups. The ITS spanned six months, a time period chosen to correspond with the semester structure of the TUSM MD program. Participant data was collected monthly for three months prior to delivery of a bibliotherapy intervention, and again for three months following. Data points for the ITS were collected using the 10-Item Perceived Stress Scale (PSS-10). Rather than building assessments based on objectively stressful life events, the PSS aims to illustrate “the degree to which situations in one's life are appraised as stressful” [24], and the instrument has been used in multiple recent studies assessing the efficacy of wellness interventions in medical student populations, although not specifically with bibliotherapy [25], [26], [27].

The PSS-10 consists of ten questions, half of which are phrased positively, and half phrased negatively. It is completed by the test subject, who reports their own reactions and stress levels over the prior month. The PSS-10 is designed as a straightforward 5-point Likert-scale with response options ranging from 0 = “Never” to 4 = “Very often”. Questions 1, 2, 3, 6, 9, and 10 are calculated as-written (0= “Never” for 0 points to 4 = “Very often” for 4 points). Question 4, 5, 7, and 8 are reverse-coded (0= “Never” for 4 points to 4 = “Very often” for 0 points) [28]. The final score is determined by adding the results of all 10 questions [29]. Scores can range from 0 to 40, with higher scores indicating higher levels of perceived stress [24]. The PSS-10 was selected for this study because it is brief, easy to understand, and widely used with a variety of populations in both clinical and empirical research settings [28]. All participants who successfully completed PSS-10 surveys were then offered the opportunity to enroll in a focus group. Successful completion of the study was defined as completing the bibliotherapy intervention and at least two pre- and post-reading PSS surveys.

The quantitative component of the study was conducted January – June 2021. The PSS-10 was administered monthly, six times in total. It was distributed on the first day of the month (or the closest weekday to the beginning of the month) with one week to complete and submit answers. A reminder was sent to those who had not submitted the PSS-10 after three days. The intervention was distributed after the March PSS-10, with all eligible participants receiving an additional Qualtrics survey listing six excerpts

PDFs of the excerpts were available for participants to download from Box. Readings were to be completed between March 8 and March 31, 2021. Participants were reminded twice to fill out a Qualtrics survey confirming that the readings were completed.

Following successful completion of the quantitative component of the study, participants received a $100 USD Visa gift card. All qualifying participants were invited to participate in a focus group designed to gather qualitative reflections. Questions were developed by the authors to obtain insight on participants' experience of this proof-of-concept study and to guide future development of bibliotherapy programs at HHSL. Our questions followed the “questioning route” strategy outlined by Krueger.

Questions were approved as part of IRB but were not pilot-tested ahead of the focus group meetings [30]. Focus groups were planned to consist of no more than 12 participants at one time, so multiple sessions were scheduled and held via Zoom in June-July 2021. Four focus groups were held, with attendance ranging from 2 to 5 participants. All focus group participants were entered into a raffle for an additional $200 USD Visa gift card, awarded to one participant following the completion of all groups. The focus group Questions are listed in Table 1.

**Table 1:** Focus Group Questions

Opening Prompt: “I would like to set the stage by having you think back about the last academic year; please share an area of your life where you have changed or grown in response to the challenges you have faced.”	

Q1	Think back over the last 6 months as you participated in the study. What did you enjoy?
Q2	Think back over the last 6 months as you participated in the study. What did you find valuable?
Q3	If you were inviting a peer or friend to participate in this project, what would you write or say to them about it?
Q4	If you were in charge of this program, what is one change you would make or what would be different?
Q5	What do you prioritize in selecting activities for wellness or self-care?

### Participants

Eligible participants consisted of all first-year students (M1) enrolled in the MD program of the Tufts University School of Medicine class of 2024. All students who started their MD or combined MD/other degree program in August 2020 were eligible, including students in the Maine Track, an MD program offered in partnership with MaineHealth. All communications with participants were maintained through a Qualtrics Contact List and all surveys and instruments distributed using Qualtrics Email Distribution. All surveys (PSS-10, Reading Selection, and Reading Confirmation) were created using the Qualtrics Anonymize Responses feature, which removes the IP address and location data from responses. The researchers could see who had responded but not which responses belonged to which participants. Preliminary research conducted by the authors indicated that medical students perceive that they may be stigmatized for disclosure of mental health conditions, or that asking for help brands them as “less successful, weak or incapable” [31]. This potential for stigma led the researchers to prioritize participant privacy, ensuring that all data collected via Qualtrics could not be associated with individuals. This choice removed the ability to compare pre-and-post intervention PSS-10 scores in pairs. While limiting our analysis, it preserved anonymity, which was determined to be essential to cultivating trust and minimizing potential harm. Participants were informed of privacy measures via a consent form they were required to authorize as part of intake and enrollment, and they were given channels to report concerns to the IRB.

Because the initial consent authorized on study enrollment noted that student responses would not be identifiable, the focus group Script contained an additional consent statement that was read before starting any recording of voice or image (see Appendix for full focus group script and consent statement). Participants had the option to leave the session before recording began. Once verbal consent was obtained from all participants, the focus groups were conducted by ALV with RM off-camera. Each focus group concluded with the opportunity for participants to ask questions about the study itself.

### Data Analysis

Following completion of six months of PSS-10 surveys, the scores for each survey were calculated according to Cohen's scoring rubric [24]. Mean scores for each month were calculated. As results of the PSS-10 were completely anonymous, we could not pair pre-and-post-intervention scores, and the groups differ in composition due to some participants being removed from the study. Because of these factors, data were analyzed using the independent samples t-test to determine differences between the population mean (pre-intervention) and the mean scores following the intervention, treating groups as mutually exclusive due to variations in makeup each month and the anonymity of participants [32]. Data were assessed using the Shapiro-Wilk test with results indicating normal distribution prior to conducting the independent samples t-test.

Focus groups were recorded to a secure hard drive and transcribed using the MacOS Advanced Dictation tool. Transcript corrections were completed by RM; names of participants were replaced with initials for identification. Transcripts were reviewed for accuracy by RM and recordings of focus group sessions were destroyed following transcription correction. Initial hand coding of transcripts was completed by ALV to identify initial themes. Transcripts were then uploaded into NVivo 1.6.2 and further analyzed using principles of the constant comparison method, a technique using open coding to identify themes within and across the different focus groups, and to reexamine already coded content as new themes emerge [33].

## RESULTS

**Figure 1:**
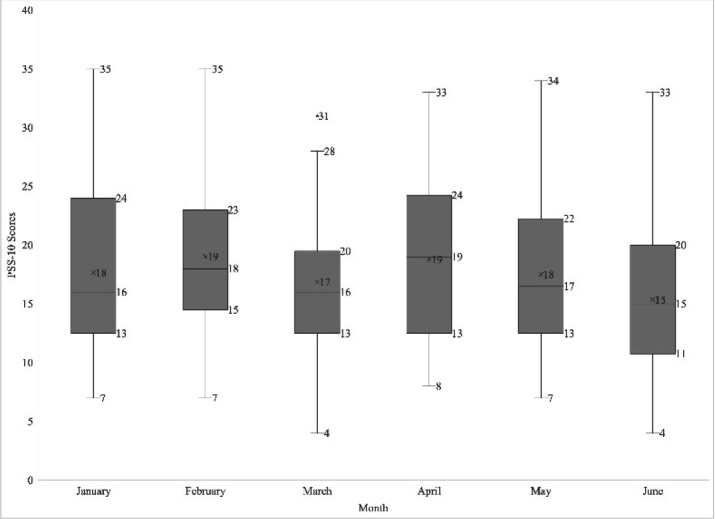
PSS-10 scores by month

We successfully recruited 30 participants (15% of the class). Of these 30 participants, 29 selected a reading and 27 completed the quantitative phase of the study. Statistical power was not calculated, rather the size of the group for this proof-of-concept pilot was based on a clinical psychology recommendation of a cohort of 25–30 participants per condition when testing empirically supported therapies [34]. Quantitative analysis was performed on 164 total complete scores, 86 pre-intervention and 78 post-intervention. The mean PSS-10 score pre-intervention was 17.85 (SD 6.76) and mean post-intervention score was 17.21 (SD 6.87) out of a possible 40. While post-intervention scores were lower, the difference was not statistically significant, t(162)= .604, two-sided p=.547, 95% CI [−1.46, 2.75]. Figure 1 shows the distribution of pooled PSS-10 scores by month.

x-value indicates the mean score for the month and the horizontal line indicates the median

Selection of bibliotherapy excerpts for the intervention was varied and all offerings were selected by more than one participant. The excerpts were a mix of fiction and non-fiction, from which participants were instructed to choose three. All excerpts were evaluated for reading time using Read-o-Meter and based on an average reading speed of 200 words per minute [35]. Selected texts were determined to require between 13 and 35 minutes to complete. The excerpts, estimated time to read, and the percentage of participants choosing each selection are listed in Table 2.

**Table 2:** Bibliotherapy Excerpts

Excerpt Name and Description	Time to Read	# of Times Selected	% of Participants who Selected the Reading
*The Diary of Samuel Pepys* (1665), an eyewitness account of the Great Plague of London	19 min	12	41.3%
*Pox Americana: The Great Smallpox Epidemic of 1775–82* by Elizabeth A. Fenn (2002), a narrative of the smallpox outbreak among Colonial troops during the American War of Independence	19 min	7	2.4%
*And the Band Played On: Politics, People, and the AIDS Epidemic* by Randy Shilts (1987), describing government and civilian reaction to the burgeoning AIDS epidemic	34 min	22	75.9%
*The Plague* by Albert Camus (1947), concerning the psychological and psychosocial impact of quarantine and isolation	17 min	20	69%
*Zone One* by Colson Whitehead (2012), a post-apocalyptic zombie novel set in New York City	13 min	17	58.6%
*Pale Horse, Pale Rider* by Katherine Ann Porter (1939), centered on the protagonist's experience of contracting and recovering from influenza in 1918	35 min	9	31%

The focus groups were analyzed to identify overall themes across the entirety of each transcript, regardless of which question the participants were responding to in the moment; for example, the identified theme of Disconnection could be identified in the answer to any individual question or in the icebreaker segment of the focus group. Through the coding process, we identified 162 discrete statements that we distilled into the following themes: Disconnection, Desire to Discuss Readings, the Fog of Medical School, and Reconnect and Reflect. Two narrower but notable themes were identified as part of Reconnect and Reflect: Value of Check-In and Self-Reliance. Reponses that related to reconnection and reflection, but not the subthemes related to checking in or self-reliance, are coded in a General subcategory. The category of Reconnect and Reflect had the most coded responses, with the majority of responses (87 statements) concerning this theme.

### Disconnection

We anticipated that feelings related to disconnection or isolation would emerge, although such sentiments made up fewer than expected coded responses (21 statements). Participants reported difficulty adjusting the demands of medical school, which were exacerbated COVID-19, but also the demands of daily living. Students attended few classes in-person in the 2020–2021 academic year, and participants noted that it was difficult to meet new people, or to get to know people outside of class. Even as restrictions eased in Spring and Summer 2021, the campus maintained limits on gatherings, constraining opportunities for socialization. Of note, several participants specifically highlighted the distance they felt from fellow medical students in different classes and feeling that they “missed out on…a lot of valuable things we learn from upperclassmen.”

### The Fog of Medical School

Twenty-four coded responses concern what we called “The Fog of Medical School,” or the general state of stress, anxiety, depersonalization, and sleep deprivation related to the rigors of MD training. Participants described their reactions to the first year of medical school in terms such as “I have a difficult time taking time for myself,” “the last couple of months have sort of been really hard, but also a blur,” “I just don't remember that any of that happened, because I'm just so focused,” and “there's so much going on that sometimes you just don't stop and think about how you feel.” Multiple participants reported that participating in the study helped with the disorientation they experienced in their M1 year. One participant noting that the study was a “reminder not to lose your interests outside of academics, outside of medicine.”

### Desire to Discuss Readings

This study was explicitly designed to eliminate the “book club” element of a traditional bibliotherapy program, as we determined that it would be impractical from a time management perspective and would be impossible when campus gathering limits remained in place due to COVID-19. In focus group discussions, 30 coded responses concerned the participants' discussion of the excerpts they selected to read for the intervention portion of the study in March 2021. Multiple participants noted confusion about what they were supposed to “do” with the readings, expressing surprise at the lack of a follow-up exam or quiz, and even concern that they had completed the task incorrectly. One participant noted that if they had realized there would be no assessment, “I would not have focused so much on content and focused more on…how it made me feel.”

Participants also noted that they wished for a forum to discuss the readings. No participants expressed an interest in the more conventional book club format, but did reference modalities such as Zoom, GroupMe, a Slack channel, or a personal journal. One participant noted that they had not thought to reflect on any effect they personally experienced while completing the intervention until the focus group. While designed to specifically exclude the discussion or meeting component common to bibliotherapy programs, it appears that participants missed the opportunity to engage with each other. Some participants noted that the content of the readings was “cool,” “interesting,” and that the readings did not feel “like a chore,” And one participant did note positive feelings about grappling with unfamiliar literature without concerns about completing a required assignment. Other participants indicated that more pandemic content and reading, in addition to the required curriculum and their regular media diet, was not welcome, and that some selections were “dense.” Several participants noted that they were glad they had a choice among the selected readings, while others suggested offering interventions in different media, such as short films or podcasts. Participants in each focus group remarked that they thought the study would require a larger time commitment with more structured work.

### Reconnect and Reflect

The thematic area “Reconnect and Reflect” was the most frequently coded throughout all transcripts. This theme refers to students' feelings of reconnection with themselves and their values, as well as their reflections on their medical school experience thus far. This thematic area accounted for 87 of the total coded responses. Multiple participants specifically noted that their participation in the study helped them remember “the enjoyment of reading” and expressed that the study forced them to read and reflect on non-curricular material. One participant expressed frustration with having so little free time for activities such as pleasure reading, noting that “medical school is basically hindering me from doing something I valued, and [I] had to rethink and reflect on that.” When speaking about reconnecting and reflecting in general, participants often noted that the first year of medical school in a pandemic forced them to reconsider existing and new relationships with family and friends, as well as their ability to entertain non-scholastic activities. Several participants emphasized the importance of friendships outside of medical school, including activities such as watching television shows via FaceTime with friends, “like we used to do in person, and it was really nice because… I never had to talk about med school.”

Participants also reflected on the reality that their lives included “a limited number of people you could see or… feel comfortable getting close with and so… Yeah, I think like the quality of my relationships increased, maybe at the expense of quantity.” Participants also discussed the concept of “reflection” itself, and the difficulty of prioritizing it.

Within the “Reconnect and Reflect” thematic area, two sub-themes emerged: (1) reflection on the value gained from the act of responding to the PSS-10 survey itself; and (2) reflection on self-reliance, i.e., one's ability to handle the challenges of medical school.

The first sub-theme, *reflection on the value gained from the act of responding to the PSS-10 survey itself,* was the most prominent sub-theme and accounted for 38 responses within the “Reconnect and Reflect” thematic area. Participants overwhelmingly noted that a valuable component of the study was the act of completing the PSS-10, and that the monthly prompt to complete the survey was welcome and helpful. There was general agreement that completing the PSS-10 once per month for six months was “not too hard and they're…a good check up every month.” The fact that the PSS-10 is short and designed to be answered quickly was frequently cited as key to its utility, with one participant noting that they worried this aspect of the study would be burdensome, as “even if its intent is good, [a wellness assessment] can be more detrimental if it's not executed properly.” Another participant reported that the PSS-10 helped them “put a name to…feelings that I had and…in a way, it kind of normalized [the feelings].” Given our emphasis on building a program that respects the demands on students' time, it appears participants embraced the PSS-10. One participant reported “I think the benefits were greater than the little time I had to spend doing the survey,” and another noted “I enjoyed that part. It was like therapy.”

The second sub-theme, *reflection on self-reliance*, accounted for 15 responses within the “Reconnect and Reflect” thematic area. Participants noted that their first year of medical school forced them to become more self-reliant and confident in ways they did not anticipate. Participants discovered that they could succeed under unusual circumstances and learn to be more flexible. They reported growing more comfortable coping with uncertainty and “coming to terms with the fact that I wasn't ever going to be 100% confident.” Participants also discussed developing self-reliance through learning how to be organized and to strengthen their executive function.

34 responses did not reference either sub-theme in the “Reconnect and Reflect” thematic area but touched on the concept broadly. The following quote typifies student responses coded for the theme “Reconnect and Reflect”:

“…I think this study kind of reminded me like, mental health has always been like a big part of my life and like, kind of maintaining it…during the pandemic was like a little difficult, but I think like, the whole study, like connecting reading, which is also something I really enjoy like, with mental health specifically, like with the surveys and everything, kind of like reminds me like, yeah, your mental health is going to improve by doing things such as reading and like other things that you enjoy. [nodding from participants] So, I think like the study kind of reached out to other points in my life that also connected back to making you feel better in your daily life.”

## DISCUSSION

Our study found that a self-paced librarian-directed bibliotherapy program may help medical students with feelings of anxiety, stress, and isolation. Although there was not a statistically significant difference in PSS-10 scores, focus group feedback indicated that students found completing the monthly assessment was beneficial for monitoring their mental health. The high incidence of depression, anxiety and suicidal ideation in health care professionals and trainees is recognized as a threat to the well-being of these workers and a risk to the reliable provision of high quality care and to patient safety [36]. Studies indicate that medical students suffering from depression are unlikely to seek treatment [37]. Both AAMC recommendations and LCME standards emphasize that wellness programming and accessible mental health services are crucial aspects of successful medical education [38], [39]. Feelings of loneliness and isolation were commonly reported among medical students and health professionals prior to the COVID-19 pandemic [40]. Research indicates that medical students experienced increased levels of loneliness in the early months of COVID-19 as compared to pre-pandemic [41]. Our observations related to Disconnection, particularly as it relates to building relationships with other medical students, seem to agree with these findings. Further, our participants' reports of anxiety and stress related to isolation, workload, and adjustment to medical school correspond to previous research [2].

While several systematic reviews attempt to assess the efficacy of interventions on health care worker and medical student well-being during the COVID-19 pandemic, there is an acknowledged lack of robust literature related to student support [42] and to the use of bibliotherapy specifically as an intervention among healthcare workers [43]. Our study suggests that in our population, the bibliotherapy intervention may have less meaningful impact as a measure to mitigate stress and anxiety than the monthly wellness surveys themselves. The study had a high rate of completion (90%) and focus group feedback identified that the PSS-10 was a useful tool for self-monitoring well-being. Participant responses referencing the PSS-10 and the utility of monthly reminders lend support to the idea that deadlines are motivational in bibliotherapy programs, even in the absence of regular therapist interaction [16]. One unanticipated finding is that several participants specifically noted a desire to see their PSS-10 scores over the course of the study and how their scores compared to others in their class. This was not possible due to the practice of anonymous data collection, implemented specifically to protect participants from possible harm related to potential exposure of poor mental health status. In addition to revealing the inherent value of this check-in, several focus group participants highlighted that they appreciated the involvement of HHSL librarians. While TUSM provides a great deal of outreach related to mental health, participants reported that our programming was “low stress…easier to access…probably more helpful” than more formal counseling efforts. This role of the library in medical student wellness is supported by the AAMC recommendation that mental health intervention is best undertaken “by different individuals than those rendering advancement or promotion decisions” [38]. This study may signal that students trust the library and librarians, supporting further development of a bibliotherapy program with less anonymity and greater ability for students to follow their own progress. Positive feedback related to readings and use of the survey instrument indicate that this proof-of-concept study was successful, and that first-year medical students derive benefit from a librarian-led bibliotherapy program.

### Limitations of the Study

Limitations of the study include the brief six-month duration of the quantitative component of the program; the interrupted time series model requires more data points to avoid seasonality and build a foundation for robust and informative analysis to determine if the intervention itself is correlated with lower PSS-10 scores. The inability to achieve statistical significance in our quantitative analysis may be attributable to the short study duration, and in addition, the fully anonymous nature of data collection, designed to protect the identities of participants in the most complete manner possible, rendered us unable to conduct a paired statistical analysis to determine intra-participant changes before and after the bibliotherapy intervention. A longer interrupted time-series and linear regression analysis or a deidentified (as opposed to anonymous) data collection process followed by paired testing would each result in more robust analysis and thus a greater indication of association between the intervention and the results.

Other limitations include that the focus groups were not subject to pilot-testing and that there is the potential for some questions to appear leading; in particular, the third question, “If you were inviting a peer or friend to participate in this project, what would you write or say to them about it?” While designed to gather insight on student perceptions of the project, this also assumes that participants would in fact recommend that a peer participate, without the obvious option of stating that they would not advise someone to do so. These untested and potentially leading questions provide some useful feedback to the researchers, but cannot be considered unbiased feedback. Because focus groups were not subject to pilot-testing, we did not have the opportunity to receive early feedback that could have prompted question revision to better yield the information we hoped to elicit. Additionally, the focus groups were conducted entirely over Zoom. While convenient, use of Zoom does introduce a potential barrier to robust discussion, especially noting the “Zoom fatigue” associated with the rapid move to online learning in March 2020 and its deleterious effects on students and instructors [44]. In addition, the participants were limited to one cohort of students at one medical school, making generalization of findings difficult. Although none of the participants had direct interactions with either author involving graded coursework, it is possible students felt inhibited from speaking freely with librarians as authority figures. Finally, the two researchers who designed and executed the study also facilitated/observed the focus groups, which could have led to observer bias, particularly in the coding and interpretation of focus group transcripts.

### Conclusion

The COVID-19 emergency brought unprecedented disruptions into everyday life. Students starting medical school in 2020 were forced to balance pandemic pressures with the substantial stress and emotional upheaval that accompanies physician training. The Hirsh Health Sciences Library responded by serving as a resource, not only for academic support, but also for self-reflection and growth. The Library has worked hard to establish its physical setting as both an academic resource and a venue for activities focused on student well-being [45]. The results of this study demonstrate that HHSL can increase its role in the student wellness landscape of the School of Medicine. While the quantitative results of this study are not statistically significant, the PSS-10 scores and results of the focus groups may indicate that medical students can derive benefits related to anxiety, stress, and isolation from a self-paced and librarian-directed bibliotherapy program. A somewhat unexpected finding is that students found completing the monthly PSS-10 itself, regardless of the intervention, to be of benefit when monitoring their own mental health. The administration of that survey by the Library, which occupies an important place outside of the grading and advancement structure of the medical school, also appears to be a factor in the students' embrace of the instrument. The bibliotherapy intervention described in this article is an example of how we can build upon established trust to provide the most important information students can have, which is information about themselves. According to the AMA Code of Ethics, “[m]edicine as a profession should continue to refine mechanisms for assessing knowledge and skill and should develop meaningful opportunities for physicians and physicians in training to hone their ability to be self-reflective and attentive in the moment.” [46] The Hirsh Heath Science Library's bibliotherapy intervention is an example of how libraries can provide “meaningful opportunities” for students to cultivate self-awareness [46].

## Data Availability

Data associated with this article are available in the Tufts University Dataverse: https://doi.org/10.7910/DVN/LQLRCD.

## Supplemental Files

**Appendix A:** Focus Group Scripts
