# Supplementary material for: Bibliotherapy for stress management: a wellness intervention for first-year medical students
Source: J Med Libr Assoc. 2025 Apr 18;113(2):158–67. doi: 10.5195/jmla.2025.1830 (PMC12058346; doi:10.5195/jmla.2025.1830)
Supplement: Supplementary file 1 — Appendix A: Focus Group Scripts [file jmla-113-2-158-s01.docx]

**FOCUS GROUP SCRIPT**

**Prior to pressing “record” in Zoom:**

Hello, my name is [redacted], and I am a librarian and researcher from [redacted]. As part of the research study on bibliotherapy that you just completed, I would like to involve you all in this focus group so that you may reflect on your feelings about the study, and I can gather some additional data. It will take about one hour of your time.

While you all signed a consent form at the outset of the study, I would like to confirm that you are still interested in participating. It’s your decision, and there are no consequences to saying no. I don’t anticipate any major risks to participation, but if at any time you want to stop participating, or if you don’t want to start at all, please tell me.

Because of the General Data Protection Regulations (EU GDPR and UK GDPR), you must be located outside the European Economic Area and outside the United Kingdom in order to participate in this focus group. Please let me know if you are in Europe or the UK so you can leave the meeting before we start recording.

We are joined today by [redacted], the researcher you have been interacting with throughout the Bibliotherapy study. She will be taking notes and monitoring technical aspects of the Zoom session. If any questions about the research study arise during the focus group, I may defer to[redacted], who will be happy to answer them later in the session.

With your permission, we would like to record this Zoom session. We will use local software to transcribe this session when we are done. If you want to answer a question but do not want it recorded on audio or video, please feel free to follow up after this session via email.

The content of our conversation may be used in publications or presentations. I will not share identifiable information about you beyond myself and people helping me with this study whom I trust to maintain your confidentiality. I will do everything I can to protect your privacy, but there is always a slight chance that someone could find out about our conversation. People responsible for monitoring this research may be able to access the data. This includes the [redacted] IRB.

I’ll also drop a link in the chat with my contact information and the contact information of the research oversight board at [redacted], the [redacted] IRB, if you need to get in touch about this research at any point in the future ([redacted])

In a moment I will ask you each in turn if you agree to participate in this focus group, and to talk to me about your experiences in the bibliotherapy study**. I’m hitting “record” now to make a record of your answers.**

- Do you agree to participate?
- Do you agree to let me record our conversation? [If the participant answers no, ask them to leave the Zoom]
- Do you agree to be quoted directly, without your name or other identifying information used, in presentations and publications?

I would like to set the stage by having you think back about the last academic year; please share an area of your life where you have changed or grown in response to the challenges you have faced. I am happy to go first…[redacted shares]

1. Think back over the last 6 months as you participated in the study. What did you enjoy?
2. Think back over the last 6 months as you participated in the study. What did you find valuable?
3. If you were inviting a peer or friend to participate in this project, what would you write or say to them about it?
4. If you were in charge of this program, what is one change you would make or what would be different?
5. What do you prioritize in selecting activities for wellness or self-care?
